# Stability of Differences in Weight-Related Characteristics of Mothers across Economic, Cultural, Social, and Environmental-Health Indicators of Socioeconomic Status

**DOI:** 10.3390/ijerph16203866

**Published:** 2019-10-12

**Authors:** Virginia Quick, Kaitlyn M. Eck, Colleen Delaney, Ryan Lewis, Carol Byrd-Bredbenner

**Affiliations:** Department of Nutritional Sciences, Rutgers University, New Brunswick, NJ 08520, USA; vquick@njaes.rutgers.edu (V.Q.); colleen.delaney@rutgers.edu (C.D.); rlewis398@gmail.com (R.L.); bredbenner@sebs.rutgers.edu (C.B.-B.)

**Keywords:** nutrition, weight, public health, economic capital, social capital, cultural capital, environmental capital, socioeconomic status

## Abstract

This study explored the differences in weight-related characteristics when socioeconomic status (SES) was assessed by economic, cultural, social, and environmental-health capital individually and as a composite with the goal of determining the stability of differences across types of capital and to ascertain whether single or a combination of capital indicators of SES should be used in nutrition and public health studies. Mothers (n = 557) of young children completed a survey assessing capital and weight-related characteristics. Mothers with higher economic, cultural, and social capital and composite SES had fewer sugar-sweetened beverage servings, fewer meals in front of the TV, more food security, and greater neighborhood space/supports for physical activity than comparators. Few differences occurred among environmental-health capital groups. Composite SES performed similarly to individual economic, cultural, and social capital measures. Findings suggest single SES indicators may be sufficiently stable to capture differences in weight-related characteristics. Each capital type captures a unique aspect of SES; thus, assessing an array of capital types could advance understanding of SES aspects on weight-related characteristics.

## 1. Introduction

Socioeconomic status (SES) is a construct that describes the rank or position of individuals, families, households, neighborhoods, or other aggregates within a society with respect to their capacity to access desired resources (e.g., goods, services, education, health care, employment, power, friendships) that, in turn, confer opportunity, power, and control [1,2,3]. SES is typically thought of as a gradient that only includes economic capital (i.e., income, wealth) [3,4,5]. However, others have expanded this traditional unidimensional view of “capital” to incorporate contributions of cultural, social, and environmental-health capital to the SES gradient [6,7,8].

Cultural capital, also called human capital, is knowledge that guides individuals or groups through society and affects interactions with others, judgment by others, and opportunities available [6,8,9,10]. Cultural capital takes the form of material goods (e.g., books, “proper” clothing) and symbolic elements (e.g., knowledge of societal customs, manners, and values; skill set; formal education credentials; socialization to express society’s valued attitudes and behaviors) that are admired and rewarded by society (e.g., college degree, slenderness). Those with more cultural capital (e.g., greater educational attainment, graduate of an elite university) have a greater opportunity to successfully navigate society and realize opportunities (e.g., better health care, better jobs), which builds more cultural capital as well as social and economic capital [11].

Social capital is access to resources arising from instrumental relationships forged through membership in a network (e.g., family, neighborhood, workplace, profession, club) [6,12,13]. Network membership confers access to people who can provide actual resources (e.g., information, employment, professional advice) or potential resources (e.g., reference letter, job leads) that individuals can use to achieve goals [14,15,16,17,18]. Placement in the societal hierarchy is a powerful determinant of individual social capital [15,19]. Thus, social capital, as an individual instrumental asset, frequently is measured by occupational prestige [19,20]. That is, some occupations (e.g., executives, managers, physicians, judges) garner greater admiration and broader networks with more resources thereby offering more social capital than less prestigious occupations (e.g., laborers such as dish washers and trash collector) [3,12,19]. Individuals in high prestige occupations have more social capital and greater access to resources than others, which may build economic and cultural capital [8].

The environment includes factors in the natural and built environment external to humans [7]. The environment, reflective of the socioeconomic conditions of an area, confers capital by allowing some activities (e.g., shopping in supermarkets or farm markets) or discouraging others (e.g., no sidewalks deter walking). Environmental health as a field addresses environmental factors that may affect health behaviors and outcomes [21,22]. By extension, environmental-health capital (also called neighborhood social capital [23]) can be conceptualized as factors in the neighborhood and community where a person lives that affect health-related choices and outcomes. Those living in neighborhoods with access to safe, well-maintained, health-supportive resources have more environmental-health capital than those residing in areas with limited access to healthy foods, poor supports for exercise, and crowded, unsafe, and run-down housing [24,25,26,27,28,29,30,31,32,33,34,35]. Environmental-health capital tends to be closely linked to economic capital and access to social and cultural capital [35].

Economic, cultural, social, and environmental-health capital begin to accrue at birth and synergistically and simultaneously build, interact, and shape one’s worldview or sense of “how things ought to be done [6,36,37].” As such, internalization of this capital affects the mental “logics” that drive one’s everyday decisions and behaviors within his or her myriad social milieus (e.g., family, neighborhood, community, workplace) as well as outward symbols of this internalization (e.g., food, clothing, school choices).

The relationship of SES to nutrition-related behaviors and health outcomes has been the focus of many studies [20]. For example, families with lower levels of educational achievement consume significantly more sugar-sweetened beverages than families with more education [38]. Children of low SES parents are significantly less healthy and more socially disadvantaged, which may limit educational opportunities and reduce the likelihood of attaining high SES and good health as adults [39]. Those residing in disadvantaged neighborhoods often have less access to recreation facilities and good quality nutrient-dense foods, greater access to fast-food restaurants, and an increased risk for obesity [34]. The three preceding examples describe cultural, social, and environmental-health capital differences, respectively. Clearly, all forms of capital matter when it comes to nutrition and health behaviors and outcomes. 

There are numerous ways to measure capital (or SES); each has its shortcomings and advantages, and there is little consensus about how to best measure it. Proxy or indicator data are typically used because numeric measures of household income frequently are not available, considered unreliable, or fail to fully represent actual wealth [3,40,41]. Common SES indicators used in research are possessions, poverty (e.g., participation in food assistance programs), educational attainment, occupational standing, or residential area [1,20,42,43]. 

Individual proxy variables for SES are expedient in research but cannot take into consideration the full array of capital available to individuals and may lead to conflicting findings [44,45]. For example, in a review of studies examining SES and weight change in adults, researchers found a consistent inverse relationship between weight gain and SES when occupation status was the SES proxy, but the relationship was less consistent when educational attainment and income were proxies [46]. An extensive review of 144 published studies indicated a strong inverse relationship between obesity and SES in women residing in developed countries [11], but a follow-up two decades later, showed less striking results, perhaps because the type and number of SES indicators used varied across studies [43]. In fact, others reported that cultural and economic capital were independent predictors of eating behaviors [40].

Despite the different contributions of economic, cultural, social, and environmental-health capital, health-related studies tend to measure only a single type of capital and represent it as overall “SES.” Using a single SES proxy variable may lead to unreliable and/or unstable results, hence a composite SES indicator based on an array of proxies may reduce measurement errors and conflicting results across studies [3]. The objective of this study was to use an existing data set to explore differences in weight-related characteristics when SES was assessed by each type of capital (i.e., economic, cultural, social, and environmental-health) individually and as a composite measure with the goal of determining the stability of differences across types of capital and to ascertain whether a single type of capital or a combination of capital indicators of SES should be used in nutrition and public health studies.

## 2. Materials and Methods

This dataset employed was the baseline cross-sectional survey data collected when participants began participation in a nutrition communication/health promotion program designed to help parents of young children shape home environments and lifestyles to support optimal child growth and healthy body weights. Baseline data were collected prior to randomization and receipt of intervention materials. The Institutional Review Board at the authors’ university approved the study, and all participants gave informed consent. A detailed report of the study protocol, sample and recruitment, and study instruments is reported elsewhere and is summarized below (Blinded for Review).

### 2.1. Sample

All participants were the primary family food gatekeeper, parents of a child aged 2–9 years, aged 20–45 years, and residents of catchment states (New Jersey or Arizona in the United States). Participants completing the baseline survey were compensated $15. Of the 605 participants completing the baseline survey online, those who were female and English speaking (N = 557) were retained as the analytic sample for this secondary analysis study to increase the homogeneity of the sample and avoid potential confounding effects of gender and non-English speakers [4,18,47,48].

### 2.2. Measures

The baseline survey dataset contains sociodemographic data (e.g., age, race/ethnicity), mother’s height and weight used to calculate body mass index (BMI), and indicators of SES and capital (e.g., mother’s own and spouse/partner’s education and occupation, zip [i.e., postal] code of her home residence). It also included scales assessing weight-related variables organized according to a socio-ecological framework (i.e., intrapersonal, interpersonal, and environmental variables). 

#### 2.2.1. SES and Capital Measurements

Economic capital was measured using the Family Affluence Scale (FAS) [49,50,51]. This widely used scale is sensitive in discriminating among affluence levels [50]. Those scoring 0 to 3, 4–6, and 7–9 were categorized as having low, middle, and high economic capital, respectively.

Educational attainment is a form of cultural capital [6,43]. Thus, the highest level of education achieved by the mother and her spouse or partner was combined to form the cultural capital proxy. High school diploma or less, some post-secondary education, and a baccalaureate degree or higher were scored 1, 2, or 3 points, respectively. In single-parent households, the absent partner’s score was 0, as recommended by others, because the partner was not present to confer cultural capital [4]. Those scoring 1 to 2, 3–4, and 5–6 were categorized as having low, middle, and high educational capital, respectively.

Occupational prestige represents social capital because it is considered the most important contributor to household social status and offers a way to quantify potential access to social capital [17,18,52,53]. Occupational prestige is based on the concept that some occupations garner greater societal respect and access to resources than others [18,54,55]. Occupational prestige rankings are assigned using the consensus of prestige ratings by a representative sample [54,55,56]. These rankings represent socioeconomic distances between occupations. Given the consistent relationship between one’s own occupational prestige and that of his or her social network and that both a mother and her spouse/partner affect children’s occupational mobility [18,57,58], occupational prestige for the mother and her spouse/partner were combined to create the social capital measure. The household prestige score was calculated by coding occupations of the mother and her spouse/partner using the US Census Bureau 2010 Occupation Code List [59] and then categorizing them using a prestige rankings score of 1 to 14 based on relative socioeconomic positional differences of occupations [18,54,55,60]. Occupational prestige ranking was awarded as shown in the Appendix A. In single-parent households, the absent spouse/partner’s score was 0, as recommended by others [4,48]. Those scoring 1 to 10, 11–20, and >20 were categorized as having low, middle, and high social capital.

Environmental-health capital was based on US Census Bureau zipcode data for variables associated with physical and mental health parameters—average community income, number of supermarkets, population density, and percent owner occupied housing. For instance, higher community income, more supermarket availability, lower population density, and higher owner-occupied housing are associated with better health, quality of life, and neighborhood safety and stability [61,62,63,64]. One point was awarded to each variable when the value for the home residence’s zipcode was at or above the median threshold for the participant’s state (NJ or AZ) of residence or 0 points when below the median. Scores of 0 to 1, 2, and 3–4 were categorized as having low, middle, and high environmental-health capital, respectively.

Low, middle, and high rankings for each type of capital (economic, cultural, social, environmental-health) were assigned a value of 0, 1, or 2, respectively. Ranking values were summed to create a composite SES indicator score (score range 0 to 8). The composite score extends the capital construct proposed by Oakes and Rossi by including environmental-health capital [3,8]. Tertiles of the composite SES indicator were computed to assess differences among the low, middle, and high composite SES indicator groups.

#### 2.2.2. Weight-Related Characteristics

Table 1 reports Cronbach’s alpha coefficients, possible score range, and score interpretations for all scales. Detailed information on the scales is published elsewhere [65,66]. In brief, intrapersonal characteristics included mothers’ overall health and weight-related behaviors. Questionnaires assessed health quality of life [67,68] and stress level [69]. Dietary Restraint was evaluated with Three-Factor Eating Questionnaire items [70,71,72]. Daily servings of fruits/vegetables [73,74,75,76,77] and a sugar-sweetened beverage [78] were also assessed. Other measures were physical activity level [79,80], sedentary screen-time [81], and usual sleep daily duration [82,83].

Intrapersonal characteristics focused on household interactions. These included frequency and location of family meals and household organization and interactions [84,85]. 

Home physical environment considered household food security risk [86], fruits/vegetables availability [87], and sugar-sweetened beverages availability [77,78]. The Home Opportunities for Physical Activity Checkup (HOP-Up) assessed physical activity availability and accessibility in and near homes [88]. 

### 2.3. Data Analysis

To examine differences in weight-related characteristics by types of capital, scores for economic, cultural, social, and environmental-health capital and composite SES were calculated, and participants were divided into groups for each type of capital (i.e., low, middle, and high). Descriptive statistics were calculated for each measure by capital groups. Spearman rank order correlations examined relationships among the four capital groups. To identify whether significant differences in weight-related characteristics occurred among low, middle, and high groups for each type of capital, analysis of variance (ANOVA) with Tukey post-hoc tests were conducted for continuous variables, and chi-square analyses were conducted for categorical variables. Analysis of covariance (ANCOVA) for continuous variables and generalized linear model analysis for categorical variables, controlling for mother’s race/ethnicity, and age with least significant difference follow-up tests were conducted to determine significant differences in characteristics among SES Index tertile groups. 

Benjamini-Hochberg false discovery rate (FDR), calculated using a P < 0.01, was used to control for type 1 errors [84]. This procedure indicated that the statistical threshold for significance was P < 0.004 to account for the numerous comparison tests for main effects. For post-hoc tests, significance was set at P < 0.05. Analyses were performed using SPSS software version 22.0 (IBM Corporation, Chicago, IL, USA). 

## 3. Results

Participants were 32.6 ± 5.5SD years old, were mostly white (60%) and in dual parent households (82%), and nearly half (49%) had earned at least a baccalaureate degree. As shown in Table 1, ANOVA and post-hoc tests revealed that for each type of capital, the low, middle, and high groups differed significantly from each other signifying assignment to the low, middle, and high groups was appropriate. Spearman rank order correlations among the four types of capital revealed weak relationships (r = 0.128 or = 0.393) for all pairwise comparisons, except cultural vs. social capital which had a moderate relationship (r = 0.569). These correlations suggest each type of capital is independent of the other. 

Mothers with higher economic, cultural, social, and environmental-health capital tended to be significantly older and have more education. The same was true when comparing low, middle, and high SES Index Tertile groups. Based on these sociodemographic findings, age was used as a covariate in ANCOVA models. Education level was not included as a covariate because this variable was used to calculate cultural capital and SES Index. Although race/ethnicity was not significantly different across SES tertiles, it was included as a covariate due to prior research consistently showing ethnic differences in the relationship between SES and obesity and health [5,93].

Intrapersonal weight-related characteristics results indicated that mothers with the most economic capital tended to have significantly better health quality of life than those with less. Those in the high and middle economic, cultural, and social capital groups tended to consume fewer daily servings of sugar-sweetened beverages than those in the low groups for these types of capital. As social and environmental-health capital increased dietary restraint scores tended to rise. Screentime was inversely related to cultural capital level. Similarly, in the subsample who reported height and weight (n = 221), cultural capital was inversely related to BMI. SES Index Tertile comparisons showed that, as tertiles increased from low to high, healthy quality of life and dietary restraint tended to improve significantly whereas mother’s sugar-sweetened beverage servings declined.

Among interpersonal characteristics, as economic, cultural, and social capital increased, the frequency of eating family meals in front of the television declined. Those in the middle or high economic and cultural capital groups and middle or high SES Index Tertile ate more meals at a dining table compared to their counterparts in the low group.

Analysis of environmental characteristics indicate that mothers with higher economic, cultural, and social capital and SES Index Tertile tended to have significantly more food security, greater neighborhood space and supports for physical activity, and higher perceptions of neighborhood safety. Mothers with more cultural and social capital and higher SES Index Tertile had less household availability of sugar-sweetened beverages than counterparts. Additionally, mothers with greater environmental-health capital had significantly higher perceptions of neighborhood safety. Those in the highest economic and cultural capital groups and SES Index Tertile had significantly greater fruit/vegetable availability than those in lower capital and tertile groups.

Overall, the composite SES Index performed similarly to the individual economic, cultural, and social capital analyses, with findings tending to be stable across intrapersonal, interpersonal, and home environment weight-related characteristics. There were few significant differences in weight-related characteristics among environmental-health capital groups.

## 4. Discussion

To the authors’ knowledge, this is the first study to compare relationships of weight-related intrapersonal, interpersonal, and environmental characteristics across SES gradients of economic, cultural, social, and environmental-health capital individually and as a composite SES Index. Overall, findings show across SES gradients that as capital rises, so do scores on weight-related characteristics associated with better health. 

Similar to previous findings, greater cultural capital (i.e., proxy for education attainment) was associated with numerous health-promoting behaviors and outcomes [39,94], such as lower consumption of sugar-sweetened beverages and less screentime, as well as better health quality of life, lower BMI, and more health-promoting home environments. As noted by others, educational attainment links with health may be at least partially explained by literacy, which supports access to information enabling more informed decisions about health [39,95].

Educational attainment shapes employment opportunities, a major determinant of economic capital. For instance, more educated individuals experience lower rates of unemployment [96] and higher compensation [97], which affects their ability to afford healthier (and often costlier) [98] foods and other health-related resources [50]. Indeed, in this study, mothers with higher economic capital were more food secure and tended to have lower intake of sugar-sweetened beverages and greater fruit/vegetable availability than those with less economic capital. Additionally, as found in other studies, families of lower economic capital reported less safe neighborhoods and less space and supports for active play along with scoring significantly lower on the environmental-health score [99,100]. Families with fewer economic resources, such as money for purchasing healthy foods or easy access to safe areas for physical activity, are at a health disadvantage. However, relationships within social networks (i.e., social capital) may help to counter the effects of being economically disadvantaged [101].

Increased social support is associated with better physical and mental health [102,103,104], and when social networks are socially advantaged, they enhance access to employment, housing, and other opportunities and resources that can influence health [104]. In this study, mothers with the lowest household occupational prestige (i.e., proxy for individual social capital) had less food security, engaged in fewer health-promoting activities, and had more availability of sugar-sweetened beverages than those with higher household occupational prestige. Mothers with ready access to knowledgeable people within their social networks may receive valuable health information, employment opportunities, and emotional supports that enable them to somewhat overcome effects of limited economic resources. Those in occupations with greater prestige may embody health-supportive lifestyle behaviors (in line with their occupational hierarchy) while also interacting with a workplace environment reinforcing healthy behaviors. Hence, it is not only who you know within your social network, but the values and social norms supportive of shaping healthy behaviors that may influence health choices and outcomes.

Research suggests that neighborhood conditions (i.e., environmental-health capital) may attenuate the effects of low economic capital, and therefore may be an important determinant of health compared with other individual SES proxies [39]. For instance, well-educated individuals living in an economically disadvantaged community likely have the health knowledge to successfully navigate the poor food environment often found in low income communities [105]. Conversely, those with low economic capital may benefit from living in higher affluence communities where they have better healthcare opportunities, access to healthy foods, and opportunities for physical activity [106]. In the study reported here, some weight-related behaviors and outcomes, such as eating in front of the television and sugar-sweetened beverage intake and household availability of these drinks, tended to differ by economic, cultural, and social capital group, but not environmental-health capital group suggesting that where you live and the associated neighborhood conditions may not play a role in these differences or its role is not as profound as other types of capital. 

The lack of relationships among environmental-health capital and weight-related characteristics is congruent with other research reporting small associations between environmental indicators of SES and personal indicators [1]. This finding may be due to the methodology used to measure environmental-health and/or the need to take previous residential locations into account to render a more complete life course view of environmental-health capital [1]. Although this study used four zipcode derived variables to determine environmental-health capital, unlike many studies which use a single variable, US zipcode data may be too blunt and/or too limited in availability of variable types to adequately determine group assignment and result in non-differential misclassifications and bias toward the null [107,108]. Additionally, environmental-health capital scores did not consider racial mix or mothers’ race/ethnicity, which may be an important factor to consider in future research as racial residential segregation may be a key mechanism perpetuating social disadvantage [109]. That is, ethnic minorities living in poorer neighborhoods may have fewer healthier food and physical activity options [110]. Indeed, a larger percentage of non-white participants in this study were in the middle environmental-health capital group than the high group. Overall, findings suggest that environmental-health capital as defined in this study may not be as potent of an influence on weight-related characteristics as other types of capital. 

For the most part, composite SES Index and individual capital analyses revealed mothers with more capital tended to have lower BMIs and greater dietary restraint than comparison groups. Thinness is often a more highly sought after trait for women in high affluence communities, which may explain the lower BMIs and greater dietary restraint [43,95]. Additionally, compared to research among US adults [111], mothers in this study with less capital consumed more sugar-sweetened beverages. Establishing a routine of eating healthy family meals, specifically at the dining table instead of in front of a TV, can be challenging for low income households, especially if parents are single and/or work shifts or multiple jobs [112]. 

The similar performance of the composite SES Index and individual economic, cultural, and social capital measures and their stability across weight-related characteristics suggests that a single SES indicator (e.g., economic, cultural, or social capital) may be sufficiently stable to capture differences in weight-related characteristics. The lack of differences noted in weight-related characteristics by environmental-health capital runs counter to the idea that the environment has important links to health and weight-related choices and behaviors [113,114,115,116]. Future research should consider other measures of environmental-health capital that may be more reflective of weight-related decisions and behaviors. 

The question remains, however, which measure of capital would promote more accurate comparisons of findings across studies and more consistent outcomes across studies? Given that (a) economic, cultural, social, and environmental-health capital each have a different contribution to SES, (b) some differences did exist among levels of capital, (c) all types of capital measured are relatively efficiently to measure using proxies, and (d) measured types of capital were not highly correlated with each other—it seems that assessing an array of types of capital in future studies could advance our understanding of “SES” links to weight-related characteristics as well as improve the ability to compare outcomes across studies.

The strengths of this study include the large and diverse sample, multiple weight-related intrapersonal, interpersonal, and environmental weight-related characteristics studied, and comparison of four types of capital and a composite index. A limitation of the social and cultural capital scores is the assumption that an absent spouse did not contribute at all to these types of capital. Future research should consider a more nuanced approach to ascertain the contributions absent spouses may play. This study also is limited by its cross-sectional nature, and the ability to generalize to populations beyond the mothers in this study is unknown. Future research should further explore differences among types of capital, alternate ways to measure types of capital (especially environmental-health capital) and construct composite SES indexes, along with studying more diverse participants. 

## Figures and Tables

**Table 1:** Comparison of socio-demographic and weight-related characteristics (grouped by intrapersonal, interpersonal, and environmental characteristics) by level of economic, cultural, social, environmental-health capitals and SES index tertile (N = 557).

Characteristic	Economic Capital Level £			ANOVA *or χ^2^	Cultural Capital Level			ANOVA *or χ^2^	Social Capital Level			ANOVA *or χ^2^
	Low (n = 78)	Middle (n = 328)	High (n = 151)	*P*	Low (n = 114)	Middle (n = 190)	High (n = 253)	*P*	Low (n = 168)	Middle (n = 223)	High (n = 166)	*P*
	Mean ± SD or N (%)	Mean ± SD or N (%)	Mean ± SD or N (%)		Mean ± SD or N (%)	Mean ± SD or N (%)	Mean ± SD or N (%)		Mean ± SD or N (%)	Mean ± SD or N (%)	Mean ± SD or N (%)	
**Socio-Demographic Characteristics**												
Age (years)	29.90 ± 5.80	32.56 ± 5.37	34.25 ± 5.16	<0.001 ^ABC^	29.95 ± 5.86	31.76 ± 5.71	34.53 ± 4.49	<0.001 ^ABC^	30.18 ± 5.62	33.25 ± 5.38	34.33 ± 4.76	<0.001 ^AB^
Race/Ethnicity				0.020				0.003				0.012
White, non-Hispanic	38 (11.38)	194 (58.08)	102 (30.54)		53 (15.87)	116 (34.73)	165 (49.40)		97 (29.04)	122 (36.53)	115 (34.43)	
Non-white and/or Hispanic	40 (17.94)	134 (60.09)	49 (21.97)		61 (27.35)	74 (33.18)	88 (39.46)		71 (31.84)	101 (45.29)	51 (22.87)	
Family Affluence Score [49,50,51] £	2.56 ± 0.68	5.16 ± 0.80	7.59 ± 0.70	<0.001 ^ABC^	4.18 ± 1.56	5.28 ± 1.67	6.16 ± 1.50	<0.001 ^ABC^	4.77 ± 1.73	5.47 ± 1.72	6.13 ± 1.49	<0.001 ^ABC^
Education Level £	4.00 ± 1.51	4.55 ± 1.46	5.38 ± 0.98	<0.001 ^ABC^	1.81 ± 0.40	3.55 ± 0.50	5.66 ± 0.47	<0.001 ^ABC^	3.82 ± 1.35	4.74 ± 1.41	5.53 ± 0.93	<0.001 ^ABC^
Household Prestige Score [18,54,55,60] £	9.67 ± 6.96	14.70 ± 8.37	18.36 ± 7.76	<0.001 ^ABC^	6.61 ± 5.44	13.01 ± 6.76	20.24 ± 6.87	<0.001 ^ABC^	4.97 ± 3.28	14.66 ± 2.33	25.56 ± 2.63	<0.001 ^ABC^
Environmental-Health Score £	2.05 ± 0.75	2.32 ± 0.86	2.60 ± 0.91	<0.001 ^ABC^	2.03 ± 0.73	2.30 ± 0.88	2.55 ± 0.88	<0.001 ^ABC^	2.19 ± 0.86	2.41 ± 0.91	2.46 ± 0.81	0.007 ^AB^
Body Mass Index (BMI) #	30.74 ± 9.22	28.72 ± 6.79	26.39 ± 5.60	0.017	31.42 ± 8.66	29.28 ± 6.75	26.57 ± 5.80	<0.001 ^BC^	29.02 ± 7.07	28.34 ± 7.30	27.69 ± 6.25	0.546
**Weight-Related Intrapersonal Characteristics**												
Health Quality of Life [67,68] ∆	5.06 ± 6.68	3.77 ± 5.34	2.77 ± 4.32	0.00 8 ^B^	5.24 ± 7.01	3.73 ± 4.84	2.93 ± 4.65	<0.001 ^AB^	4.63 ± 6.18	3.31 ± 4.81	3.20 ± 5.00	0.020
Perceived Stress [69] (α = 0.78) ‡ ¶	3.29 ± 0.79	3.38 ± 0.78	3.52 ± 0.67	0.051	3.30 ± 0.87	3.36 ± 0.75	3.49 ± 0.70	0.048	3.35 ± 0.84	3.42 ± 0.71	3.45 ± 0.73	0.448
Dietary Restraint [70,71,72] (α = 0.72) ¶	2.32 ± 0.83	2.38 ± 0.71	2.55 ± 0.64	0.020	2.30 ± 0.83	2.37 ± 0.72	2.51 ± 0.65	0.014	2.27 ± 0.75	2.44 ± 0.72	2.55 ± 0.65	0.001 ^AB^
Fruits/Vegetables (servings/day) [73,74,75,76,77]	4.55 ± 1.98	4.31 ± 1.90	4.70 ± 1.76	0.092	4.67 ± 2.08	4.32 ± 1.93	4.45 ± 1.74	0.286	4.61 ± 2.07	4.22 ± 1.82	4.60 ± 1.73	0.063
Sugar-Sweetened Beverages (servings/day) [78]	1.06 ± 0.99	0.73 ± 0.83	0.57 ± 0.71	<0.001 ^AB^	1.13 ± 0.98	0.81 ± 0.82	0.49 ± 0.70	<0.001 ^ABC^	0.92 ± 0.93	0.69 ± 0.75	0.61 ± 0.83	0.002 ^AB^
Physical Activity Level [79,80] (α = 0.71) ¥	15.55 ± 10.43	13.64 ± 9.74	14.81 ± 9.57	0.208	15.26 ± 10.40	13.37 ± 8.93	14.40 ± 10.14	0.248	15.28 ± 9.75	13.49 ± 9.75	14.15 ± 9.90	0.201
Screen-time Activity (minutes/day) [81]	362.3 ± 289.6	361.6 ± 284.9	320.3 ± 251.1	0.292	426.7 ± 328.8	358.0 ± 269.5	310.5 ± 249.1	0.001 ^B^	387.2 ± 290.5	350.1 ± 279.9	313.7 ± 254.7	0.053
Sleep Duration (hours/day) [82,83]	7.09 ± 1.30	6.97 ± 1.27	7.29 ± 1.15	0.035	7.00 ± 1.39	7.08 ± 1.22	7.10 ± 1.21	0.794	7.08 ± 1.31	7.12 ± 1.25	6.99 ± 1.19	0.565
**Weight-Related Interpersonal Characteristics**												
Family Meal Frequency/week [89]	11.83 ± 5.15	12.96 ± 4.94	12.80 ± 4.23	0.174	12.70 ± 5.11	13.02 ± 4. 82	12.59 ± 4.64	0.638	13.47 ± 4.85	12.75 ± 5.04	12.05 ± 4.30	0.026
Location of Family Meals/week [90,91,92]												
Fast Food Restaurant	0.76 ± 1.0	0.93 ± 1.32	0.81 ± 1.12	0.384	1.02 ± 1.30	0.93 ± 1.18	0.77 ± 1.22	0.140	0.82 ± 1.06	0.90 ± 1.22	0.90 ± 1.38	0.758
In Front of TV	3.00 ± 2.69	2.05 ± 2.30	1.91 ± 2.33	0.002 ^AB^	2.89 ± 2.44	2.24 ± 2.42	1.74 ± 2.25	<0.001 ^AB^	2.60 ± 2.48	2.09 ± 2.36	1.76 ± 2.25	0.005 ^B^
At Dining Table	3.96 ± 2.63	5.03 ± 2.33	5.27 ± 2.04	<0.001 ^AB^	4.18 ± 2.60	4.99 ± 2.29	5.25 ± 2.17	<0.001 ^AB^	4.76 ± 2.40	4.78 ± 2.49	5.35 ± 1.97	0.028
Household Organization [84] (α = 0.86) §	3.71 ± 1.13	3.43 ± 1.08	3.44 ± 1.02	0.101	3.51 ± 1.25	3.34 ± 1.11	3.55 ± 0.95	0.118	3.45 ± 1.17	3.48 ± 1.07	3.48 ± 0.98	0.963
Family Conflict & Cohesion [85] (α = 0.85) §	4.00 ± 0.75	4.03 ± 0.76	4.06 ± 0.72	0.835	4.03 ± 0.79	3.93 ± 0.79	4.12 ± 0.69	0.027	4.01 ± 0.80	4.00 ± 0.71	4.11 ± 0.74	0.314
**Weight-Related Environmental Characteristics**												
Food Security Level [86] (α = 0.86) ¶	2.73 ± 1.00	3.17 ± 0.98	3.60 ± 0.68	<0.001 ^ABC^	2.73 ± 1.04	3.10 ± 1.01	3.55 ± 0.70	<0.001 ^ABC^	2.97 ± 1.01	3.26 ± 0.95	3.44 ± 0.82	<0.001 ^AB^
Household Availability												
Sugar-sweetened Beverage (serving/person/day)	0.26 ± 0.26	0.21 ± 0.22	0.19 ± 0.22	0.058	0.30 ± 0.28	0.23 ± 0.22	0.16 ± 0.20	<0.001 ^ABC^	0.26 ± 0.25	0.20 ± 0.22	0.17 ± 0.21	0.001 ^ABC^
Fruit/Vegetable (serving/person/day) [87]	5.59 ± 2.08	5.81 ± 2.08	6.57 ± 1.88	<0.001 ^BC^	5.78 ± 2.11	5.68 ± 2.14	6.30 ± 1.92	0.003 ^C^	5.90 ± 2.17	5.84 ± 2.00	6.26 ± 1.99	0.113
Home Opportunities for Physical Activity (PA) Check-Up [88]												
Indoor Home PA Space & Supports (α = 0.71) §	3.29 ± 0.92	3.36 ± 0.86	3.41 ± 0.74	0.558	3.26 ± 0.86	3.38 ± 0.80	3.36 ± 0.85	0.408	3.42 ± 0.81	3.34 ± 0.83	3.28 ± 0.87	0.326
Outdoor/Yard PA Space & Supports (α = 0.73) §	4.32 ± 0.64	4.36 ± 0.66	4.50 ± 0.64	0.058	4.34 ± 0.72	4.36 ± 0.67	4.45 ± 0.62	0.257	4.37 ± 0.73	4.36 ± 0.63	4.47 ± 0.60	0.241
Neighborhood PA Space & Supports (α = 0.89) §	3.65 ± 1.13	3.98 ± 1.01	4.38 ± 0.74	<0.001 ^ABC^	3.79 ± 1.01	3.92 ± 1.06	4.25 ± 0.89	<0.001 ^BC^	3.85 ± 1.05	4.05 ± 0.96	4.22 ± 0.95	0.003 ^B^
Neighborhood Environment Safety (α = 0.48) §	3.15 ± 0.87	3.41 ± 0.89	3.69 ± 0.78	<0.001 ^ABC^	3.22 ± 0.97	3.35 ± 0.86	3.62 ± 0.81	<0.001 ^BC^	3.29 ± 0.88	3.54 ± 0.89	3.49 ± 0.83	0.014
**Characteristic**	**Environmental-Health Capital Level**			ANOVA *	**SES Index Tertile ^**						**ANCOVA †**	
	**Low** **(n = 73)**	**Middle** **(n = 259)**	**High** **(n = 225)**	***P*** **or χ^2^**	**Low** **(n = 167)**		**Middle** **(n = 180)**		**High** **(n = 210)**		***P*** **or χ^2^**	
	**Mean ± SD or N (%)**	**Mean ± SD or N (%)**	**Mean ± SD or N (%)**		**Mean ± SD or N (%)**		**Mean ± SD or N (%)**		**Mean ± SD or N (%)**			
**SOCIO-DEMOGRAPHIC CHARACTERISTICS**												
Age (years)	31.71 ± 5.66	32.14 ± 5.47	33.53 ± 5.46	0.007 ^BC^	29.57 ± 5.51		33.30 ± 5.32		34.53 ± 4.65		<0.001 ^ABC^	
Race/Ethnicity				0.001							0.193	
White, non-Hispanic	52 (15.57)	134 (40.12)	148 (44.31)		84 (25.16)		111 (32.23)		139 (41.62)			
Non-white and/or Hispanic	21 (9.42)	125 (56.05)	77 (34.53)		83 (37.22)		69 (30.94)		71 (31.84)			
Family Affluence Score [49,50,51] £	4.41 ± 1.56	4.48 ± 1.45	5.04 ± 1.28	<0.001 ^BC^	4.05 ± 1.58		5.48 ± 1.24		6.55 ± 1.40		<0.001 ^ABC^	
Education Level £	5.19 ± 1.60	5.10 ± 1.76	5.94 ± 1.65	<0.001 ^BC^	2.44 ± 0.97		4.17 ± 1.19		5.50 ± 0.75		<0.001 ^ABC^	
Household Prestige Score [18,54,55,60] £	13.37 ± 8.24	14.41 ± 8.63	16.18 ± 8.15	0.015	7.04 ± 5.08		14.39 ± 6.38		21.82 ± 6.03		<0.001 ^ABC^	
Environmental-Health Score £	0.88 ± 0.33	2.00 ± 0.00	3.25 ± 0.44	<0.001 ^ABC^	1.85 ± 0.73		2.24 ± 0.85		2.87 ± 0.72		<0.001 ^ABC^	
Body Mass Index (BMI) #	28.46 ± 6.21	28.97 ± 7.39	27.68 ± 6.53	0.414	30.26 ± 8.29		29.02 ± 6.54		26.51 ± 5.74		0.007 ^BC^	
**WEIGHT-RELATED INTRAPERSONAL CHARACTERISTICS**												
Health Quality of Life [67,68] ∆	3.46 ± 4.59	4.08 ± 6.03	3.29 ± 4.66	0.252	4.96 ± 6.56		3.39 ± 4.69		2.90 ± 4.56		0.001 ^AB^	
Perceived Stress [69] (α = 0.78) ‡ ¶	3.29 ± 0.86	3.38 ± 0.74	3.47 ± 0.74	0.194	3.28 ± 0.84		3.41 ± 0.74		3.50 ± 0.69		0.109	
Dietary Restraint [70,71,72] (α = 0.72) ¶	2.26 ± 0.69	2.37 ± 0.72	2.53 ± 0.71	0.004 ^BC^	2.23 ± 0.79		2.48 ± 0.65		2.52 ± 0.69		<0.001 ^AB^	
Fruits/Vegetables (servings/day) [73,74,75,76,77]	4.63 ± 1.91	4.38 ± 1.87	4.47 ± 1.89	0.597	4.66 ± 2.01		4.04 ± 1.83		4.63 ± 1.76		0.002 ^AC^	
Sugar-Sweetened Beverages (servings/day) [78]	0.84 ± 0.86	0.80 ± 0.85	0.62 ± 0.81	0.024	1.09 ± 0.98		0.67 ± 0.71		0.50 ± 0.71		<0.001 ^AB^	
Physical Activity Level [79,80] (α = 0.71) ¥	13.32 ± 8.96	14.59 ± 9.80	14.10 ± 10.09	0.597	14.65 ± 10.32		14.16 ± 9.01		13.95 ± 10.07		0.978	
Screen-time Activity (minutes/day) [81]	408.1 ± 316.3	354.0 ± 277.0	327.7 ± 260.7	0.094	402.5 ± 300.8		352.3 ± 279.0		307.6 ± 248.0		0.060	
Sleep Duration (hours/day) [82,83]	6.91 ± 1.35	7.11 ± 1.26	7.08 ± 1.20	0.499	7.08 ± 1.34		6.98 ± 1.21		7.13 ± 1.20		0.509	
**WEIGHT-RELATED INTERPERSONAL CHARACTERISTICS**												
Family Meal Frequency/week [89]	11.96 ± 4.49	12.74 ± 4.95	13.04 ± 4.70	0.246	12.62 ± 5.22		13.33 ± 4.56		12.38 ± 4.62		0.112	
Location of Family Meals/week [90,91,92]												
Fast Food Restaurant	0.70 ± 0.95	0.90 ± 1.20	0.90 ± 1.33	0.421	0.89 ± 1.17		0.92 ± 1.20		0.82 ± 1.29		0.511	
In Front of TV	2.12 ± 2.44	2.18 ± 2.41	2.11 ± 2.35	0.940	2.73 ± 2.48		2.08 ± 2.39		1.73 ± 2.22		0.007 ^AB^	
At Dining Table	4.51 ± 2.53	4.84 ± 2.39	5.21 ± 2.17	0.049	4.29 ± 2.61		5.19 ± 2.16		5.25 ± 2.14		0.003 ^AB^	
Household Organization [84] (α = 0.86) §	3.23 ± 1.08	3.58 ± 1.08	3.42 ± 1.05	0.033	3.52 ± 1.20		3.38 ± 1.03		3.51 ± 1.00		0.374	
Family Conflict & Cohesion [85] (α = 0.85) §	4.02 ± 0.68	4.07 ± 0.71	4.00±0.81	0.603	3.99 ± 0.78		4.02 ± 0.72		4.08 ± 0.75		0.368	
**WEIGHT-RELATED ENVIRONMENTAL CHARACTERISTICS**												
Food Security Level [86] (α = 0.86) ¶	3.16 ± 0.97	3.22 ± 0.94	3.25 ± 0.95	0.802	2.81 ± 1.04		3.28 ± 0.91		3.50 ± 0.77		<0.001 ^ABC^	
Household Availability												
Sugar-sweetened Beverage (serving/person/day)	0.25 ± 0.26	0.22 ± 0.23	0.19 ± 0.21	0.080	0.28 ± 0.25		0.21 ± 0.22		0.16 ± 0.20		0.001 ^BC^	
Fruit/Vegetable (serving/person/day) [87]	5.70 ± 1.88	5.90 ± 2.13	6.16 ± 2.01	0.174	5.72 ± 2.07		5.72 ± 2.05		6.41 ± 1.97		0.003 ^BC^	
Home Opportunities for Physical Activity (PA) Check-Up [88]												
Indoor Home PA Space & Supports (α = 0.71) §	3.34 ± 0.90	3.30 ± 0.83	3.41 ± 0.82	0.328	3.32 ± 0.87		3.37 ± 0.81		3.35 ± 0.83		0.663	
Outdoor/Yard PA Space & Supports (α = 0.73) §	4.49 ± 0.56	4.34 ± 0.67	4.42 ± 0.66	0.188	4.34 ± 0.68		4.37 ± 0.66		4.46 ± 0.63		0.338	
Neighborhood PA Space & Supports (α = 0.89) §	4.08 ± 0.89	3.95 ± 1.00	4.13 ± 1.00	0.133	3.81 ± 0.98		3.94 ± 1.06		4.31 ± 0.89		<0.001 ^BC^	
Neighborhood Environment Safety (α = 0.48) §	3.42 ± 0.81	3.28 ± 0.90	3.64 ± 0.83	<0.001 ^BC^	3.20 ± 0.88		3.46 ± 0.89		3.63 ± 0.82		<0.001 ^AB^	

£ Family affluence score is the proxy for Economic Capital. Family Affluence Scale (FAS) [49,50,51] contains four items. Scores could range from 0–9; scores of 0–3, 4–6, and 7–9 were categorized as having low, middle, and high economic capital, respectively. Education level is the proxy for Cultural Capital. High school diploma or less, some post-secondary education, and a baccalaureate degree or higher were scored 1, 2, or 3 points, respectively. In single-parent households, the absent partner’s score was 0 because the partner was not present to confer cultural capital. Cultural capital scores could range from 1 to 6; scores of 1 to 2, 3–4, and 5–6 were categorized as having low, middle, and high educational capital, respectively. Household prestige is the proxy for Social Capital. Household prestige score was calculated by assigning occupations of the mother and her spouse/partner a prestige ranking score of 1–15 based on US Census major occupational prestige categories that describe socioeconomic differences between occupations [55]. In single-parent households, the absent spouse/partner’s score was 0 [4,48]. Household prestige scores could range from 0–30. Those scoring 1 to 10, 11–20, and 21 to 30 were categorized as having low, middle, and high social capital. Environmental-health score is the proxy for Environmental-Health Capital. US Census Bureau zipcode data for each participant’s residence for four variables (i.e., average community income, number of supermarkets, population density, and percent owner occupied housing) were each awarded 1 point the value for the home residence’s zipcode was at or above the median threshold for the participant’s state (NJ or AZ) of residence or 0 points if the value was below the median threshold. Variable scores were summed with a possible score range of 0–4; scores of 0 to 1, 2, and 3–4 were categorized as having low, middle, and high environmental-health capital, respectively. ^ SES Index was calculated by assigning low, middle, or high rankings for each type of capital (economic, cultural, social, and environmental-health) a value 0, 1, or 2 respectively and summing for a score range of 0–8. * ANOVA for continuous variables and chi-square analyses for categorical variables indicate significant (p < 0.004) main effects among economic, cultural, social, or environmental-health capital groups. † ANCOVA (controlling for age and race/ethnicity) for continuous variables and generalized linear model analyses for categorical variables indicate significant (p < 0.004) main effects among SES Index tertiles. A Tukey post-hoc tests for capital groups and least significant difference tests for SES Index tertiles indicate significant (p < 0.05) between group differences of low and middle capital and/or tertile groups. B Tukey post-hoc tests for capital groups and least significant difference tests for SES Index tertiles indicate significant (p < 0.05) between group differences of low and high capital and/or tertile groups. C Tukey post-hoc tests for capital groups and least significant difference tests for SES Index tertiles indicate significant (p < 0.05) between group differences of middle and high capital and/or tertile groups. # N = 221 (non-overweight status n = 86; overweight status n = 135). § Possible score range = 1–5; higher scores indicate great expression of the characteristic measured. ∆ Possible range 0 to 30; higher scores indicate poorer health-related quality of life. ‡ Cronbach’s alpha; not applicable if not reported in table. ¶ Possible score range = 1 to 4; higher scores indicate great expression of the characteristic measured. ¥ Possible score range 0–42; higher scores indicate more physical activity.

## Appendix A

**Table A1:** Occupational Prestige Categories *.

Occupational Prestige Category *	Score	Participant	Spouse/Partner
No paid employment	1	238	47
Handlers, equipment cleaners, helpers, and laborers	2	2	13
Transportation and material moving occupations	3	0	19
Machine operators, assemblers, and inspectors	4	2	8
Precision production, craft, and repair	5	3	65
Farming, forestry, and fishing	6	1	5
Service occupations, except protective and household	7	38	34
Protective service occupations	8	5	31
Private household occupations	9	1	0
Administrative support occupations, including clerical	10	79	33
Sales occupations	11	14	28
Technicians and related support occupations	12	42	39
Professional specialty occupations	13	106	97
Executive, administrative, and managerial occupations	14	26	34
**Total**		**557**	**453**

* Categories based on US Census Bureau occupational codes [55,59].

## References

[1] Galobardes B., Shaw M., Lawlor D.A., Lynch J.W., Smith G.D. (2006). Indicators of socioeconomic position (part 1). J. Epidemiol. Community Health.

[2] American Psychological Association Socioeconomic Status. www.apa.org/topics/socioeconomic-status/.

[3] Oakes J.M. (2012). Improving the Measurement of Socioeconomic Status for the National Assessment of Education Progress: A Theoretical Foundation.

[4] Coleman J.S. (1988). Social capital in the creation of human capital. Am. J. Sociol..

[5] Zhang Q., Wang Y. (2004). Socioeconomic inequality of obesity in the United States: Do gender, age, and ethnicity matter?. Soc. Sci. Med..

[6] Bourdieu P., Richardson J. (1986). The forms of capital. Handbook of Theory and Research for the Sociology of Education.

[7] Karol J., Gale T., Jeffrey P. (2005). Bourdieu’s social theory and sustainability: What is “environmental capital”?. Doing the Public Good: Positioning Education Research.

[8] Oakes J.M., Rossi P.H. (2003). The measurement of SES in health research: Current practice and steps toward a new approach. Soc. Sci. Med..

[9] Johnson C.L., Gutter M., Xu Y., Cho S.H., DeVaney S. (2016). Perceived value of college as an investment in human and social capital: Views of generations X and Y. Fam. Consum. Sci. Res. J..

[10] Lareau A., Weininger E.B. (2003). Cultural capital in educational research: A critical assessment. Theory Soc..

[11] Sobal J., Stunkard A.J. (1989). Socioeconomic status and obesity: A review of the literature. Psychol. Bull..

[12] Lin N. (1999). Social networks and status attainment. Annu. Rev. Sociol..

[13] Portes A. (1998). Social capital: Its origins and applications in modern sociology. Annu. Rev. Sociol..

[14] Abel T., McQueen D., Kickbusch I., Potvin L., Pelikan J., Balbo L., Abel T. (2007). Cultural Capital in Health Promotion. Health and Modernity.

[15] Adler P.S., Kwon S.W. (2002). Social capital: Prospects for a new concept. Acad. Manag. Rev..

[16] Montano D.E., Kasprzyk D., Glanz K., Rimer B., Viswanath K. (2015). Theory of reasoned action, theory of planned behavior, and the integrated behavioral model. Health Behavior. Theory, Research, and Practice.

[17] Moore S., Daniel M., Paquet C., Dube L., Gauvin L. (2009). Association of individual network social capital with abdominal adiposity, overweight and obesity. J. Public Health (Oxf.).

[18] Schulz B., Horr A., Hoenig K. (2017). NEPS Survey Paper No. 23, The Position Generator in the NEPS.

[19] Verhaeghe P.P., Li Y., Li Y. (2015). The position generator approach to social capital research: Measurements and results. The Handbook of Research Methods and Applications on Social Capital.

[20] Galobardes B., Shaw M., Lawlor D.A., Lynch J.W., Smith G.D. (2006). Indicators of socioeconomic position (part 2). J. Epidemiol. Community Health.

[21] Agius R. What is “Environmental Health”?. www.agius.com/hew/resource/envhlth.htm.

[22] World Health Organization Environmental Health. www.who.int/topics/environmental_health/en/.

[23] Sampson R.J., Graif C. (2009). Neighborhood social capital as differential social organization. Am. Behav. Sci..

[24] Barr D.A. (2014). Understanding how low social status leads to poor health. Health Disparities in the United States: Social Class, Race, Ethnicity & Health.

[25] Centers for Disease Control and Prevention (2008). Community Health and Program Services (CHAPS): Health Disparities among Racial/Ethnic Populations.

[26] Gordon-Larsen P., Nelson M.C., Page P., Popkin B.M. (2006). Inequality in the built environment underlies key health disparities in physical activity and obesity. Pediatrics.

[27] Grimm K.A., Moore L.V., Scanlon K.S. (2013). Access to healthier food retailers—United States, 2011. Morb. Mortal. Wkly. Rep..

[28] Igel U., Romppel M., Baar J., Brahler E., Grande G. (2016). The association between area-level socio-economic status and childhood overweight and the role of urbanicity. Obes. Med..

[29] Jilcott S.B., Wade S., McGuirt J.T., Wu Q., Lazorick S., Moore J.B. (2011). The association between the food environment and weight status among eastern North Carolina youth. Public Health Nutr..

[30] Logan J.E., Hall J., McDaniel D., Stevens M.R. (2013). Homicides—United States, 2007 and 2009. Morb. Mortal. Wkly. Rep..

[31] Meyer P.A., Yoon P.W., Kaufmann R.B. (2013). Introduction: CDC health disparities and inequalities report—United States, 2013. Morb. Mortal. Wkly. Rep..

[32] Navalpotro L., Regidor E., Ortega P., Martinez D., Villanueva R., Astasio P. (2012). Area-based socioeconomic environment, obesity risk behaviours, area facilities and childhood overweight and obesity socioeconomic environment and childhood overweight. Prev. Med..

[33] Rutt C.D., Coleman K.J. (2005). Examining the relationships among built environment, physical activity, and body mass index in El Paso, TX. Prev. Med..

[34] Sallis J.F., Glanz K. (2009). Physical activity and food environments: Solutions to the obesity epidemic. Millbank Q..

[35] Stafford M., Marmot M. (2003). Neighbourhood deprivation and health: Does it affect us all equally?. Int. J. Epidemiol..

[36] Power E.M. (1999). An introduction to Pierre Bourdieu’s key theoretical concepts. J. Study Food Soc..

[37] Stone D. (1988). Policy Paradox and Political Reason.

[38] Han E., Powell L. (2013). Consumption patterns of sugar-sweetened beverages in the United States. J. Acad. Nutr. Diet..

[39] Braveman P., Egerter S., Williams D.R. (2011). The social determinants of health: Coming of age. Annu. Rev. Public Health.

[40] Fismen A.-S., Samdal O., Torsheim T. (2012). Family affluence and cultural capital as indicators of social inequalities in adolescent’s eating behaviours: A population-based study. BMC Public Health.

[41] Hajat A., Kaufman J.S., Rose K.M., Siddiqi A., Thomas J.C. (2010). Do the wealthy have a health advantage? Cardiovascular disease risk factors and wealth. Soc. Sci. Med..

[42] Ezzati M., Stephen V.H., Lawes C.M., Leach R., James W.P., Lopez A.D., Rodgers A., Murry C. (2005). Rethinking the disease of affluence paradigm global patterns of nutritional risk in relation to economic development. PLoS Med..

[43] McLaren L. (2007). Socioeconomic Status and Obesity. Epidemiol. Rev..

[44] Alkerwi A., Vernier C., Sauvageot N., Crichton G.E., Elias M.F. (2015). Demographic and socioeconomic disparity in nutrition: Application of a novel correlated component regression approach. BMJ Open.

[45] Kolenikov S., Angeles G. (2008). Socioeconomic status measurement with discrete proxy variables: Is principal component analysis a reliable answer?. Rev. Income Wealth.

[46] Ball K., Crawford D. (2005). Socioeconomic status and weight change in adults—A review. Soc. Sci. Med..

[47] Song L. (2012). Raising network resources while raising children? Access to social capital by parenthood status, gender, and marital status. Soc. Netw..

[48] Westin M., Westerling R. (2007). Social capital and inequality in health between single and couple parents in Sweden. Scand. J. Public Health.

[49] Andersen A., Krølner R., Currie C., Dallago L., Due P., Richter M., Orkényi A., Holstein B.E. (2008). High agreement on family affluence between children’s and parents’ reports: International study of 11-year-old children. J. Epidemiol. Community Health.

[50] Currie C., Mollcho M., Boyce W., Holstein B., Torsheim T., Richter M. (2008). Researching health inequalities in adolescents: The development of the health behavior in school-aged children (HBSC) family affluence scale. Soc. Sci. Med..

[51] Hartley J.E.K., Levin K., Currie C. (2016). A new version of the HBSC Family Affluence Scale—FAS III: Scottish qualitative findings from the international FAS developments study. Child Indic. Res..

[52] Nock S.L., Rossi P.H. (1979). Household types and social standing. Soc. Forces.

[53] Van der Gaag M., Webber M., Kawachi I., Subramanian S., Kim D. (2008). Measurement of individual social capital. Questions, instruments, and measures. Social Capital and Health.

[54] Nakao K., Treas J. (1994). Updating occupational prestige and socioeconomic scores: How the new measures measure up. Sociol. Methodol..

[55] Christ S.L., Fleming L.E., Lee D.J., Muntaner C., Muennig P.A., Caban-Martinez A.J. (2012). The effects of a psychosocial dimension of socioeconomic position on survival: Occupational prestige and mortality among US working adults. Sociol. Health Illn..

[56] McClendon M.J. (1976). The occupational status attainment processes of males and females. Am. Sociol. Rev..

[57] Rosenfeld R.A. (1978). Women’s intergenerational occupational mobility. Am. Sociol. Rev..

[58] U.S. Census Bureau 2010 Occupation Code List. https://www.census.gov/topics/employment/industry-occupation/guidance/code-lists.html.

[59] Hauser R.M., Warren J.R. (1997). Socioeconomic indexes for occupations: A review, update, and critique. Sociol. Methodol..

[60] Lederbogen F., Kirsch P., Haddad L., Streit F., Tost H., Schuch P., Wust S., Pruessner J.C., Rietschel M., Deuschle M. (2011). City living and urban upbringing affect neural social stress processing in humans. Nature.

[61] Fassio O., Chiara R., De Piccoli N. (2013). Health, quality of life and population density: A preliminary study on “contextualized” quality of life. Soc. Indic. Res..

[62] Research Division Social Benefits of Homeownership and Stable Housing. http://www.realtor.org/sites/default/files/social-benefits-of-stable-housing-2012-04.pdf.

[63] Sundquist K., Frank G., Sundquist J. (2004). Urbanisation and incidence of psychosis and depression. Br. J. Psychiatry.

[64] Martin-Biggers J. (2016). Home Environment Characteristics Associated with Obesity Risk in Preschool-Aged Children and Their Parents.

[65] Quick V., Martin-Biggers J., Povis G., Hongu N., Worobey J., Byrd-Bredbenner C. (2017). A socio-ecological examination of weight-related characteristics of the home environment and lifestyles of households with young children. Nutrients.

[66] Centers for Disease Control and Prevention HRQOL Concepts Why is Quality of Life Important?. www.cdc.gov/hrqol/concept.htm.

[67] Centers for Disease Control and Prevention CDC HRQOL-14 Healthy Days Measure. www.cdc.gov/hrqol/hrqol14_measure.htm.

[68] Cohen S., Kamarck T., Mermelstein R. (1983). A global measure of perceived stress. J. Health Soc. Behav..

[69] Cappelleri J.C., Bushmakin A.G., Gerber R.A., Leidy N.K., Sexton C.C., Lowe M.R., Karlsson J. (2009). Psychometric analysis of the Three-Factor Eating Questionnaire-R21: Results from a large diverse sample of obese and non-obese participants. Int. J. Obes. (Lond.).

[70] Karlsson J., Persson L.O., Sjostrom L., Sullivan M. (2000). Psychometric properties and factor structure of the Three-Factor Eating (TFEQ) in obese men and women. Results from the Swedish Obese Subjects (SOS) study. Int. J. Obes..

[71] Stunkard A.J., Messick S. (1985). The three-factor eating questionnaire to measure dietary restraint, disinhibition and hunger. J. Psychosom. Res..

[72] Block G., Gillespie C., Rosenbaum E.H., Jenson C. (2000). A rapid food screener to assess fat and fruit and vegetable intake. Am. J. Prev. Med..

[73] Block G., Hartman A.M., Naughton D. (1990). A reduced dietary questionnaire: Development and validation. Epidemiology.

[74] Block G., Thompson F.E., Hartman A.M., Larkin F.A., Guire K.E. (1992). Comparison of two dietary questionnaires validated against multiple dietary records collected during a 1-year period. J. Am. Diet. Assoc..

[75] Wakimoto P., Block G., Mandel S., Medina N. (2006). Development and Reliability of Brief Dietary Assessment Tools for Hispanics. Prev. Chronic Dis..

[76] West D.S., Bursac Z., Quimby D., Prewitt T.E., Spatz T., Nash C., Mays G., Eddings K. (2006). Self-reported sugar-sweetened beverage intake among college students. Obesity.

[77] Nelson M.C., Lytle L.A. (2009). Development and evaluation of a brief screener to estimate fast-food beverage consumption among adolescents. J. Am. Diet. Assoc..

[78] Lee P., Macfarlane D., Lam T., Stewart S. (2011). Validity of the international physical activity questionnaire short form (IPAQ-SF): A systematic review. Int. J. Behav. Nutr. Phys. Act..

[79] Quick V., Byrd-Bredbenner C., Shoff S., White A.A., Lohse B., Horacek T., Kattelmann K.K., Phillips B., Hoerr S., Greene G. (2016). A streamlined, enhanced self-report physical activity measure for young adults. Int. J. Health Promot. Educ..

[80] Owen N., Sugiyama T., Eakin E.E., Gardiner P.A., Tremblay M.S., Sallis J.F. (2011). Adults’ sedentary behavior determinants and interventions. Am. J. Prev. Med..

[81] Buysse D.J., Reynolds C.F., Monk T.H., Berman S.R., Kupfer D.J. (1989). The Pittsburgh Sleep Quality Index: A new instrument for psychiatric practice and research. Psychiatr. Res..

[82] Carpenter J.S., Andrykowski M.A. (1998). Psychometric evaluation of the Pittsburgh Sleep Quality Index. J. Psychosom. Res..

[83] Matheny A.P., Wachs T.D., Ludwig J.L., Phillips K. (1995). Bringing order out of chaos: Psychometric characteristics of the confusion, hubbub, and order scale. J. Appl. Dev. Psychol..

[84] Moos R.H., Moos B.S. (1994). Family Environment Scale Manual: Development, Applications, Research.

[85] Hager E.R., Quigg A.M., Black M.M., Coleman S.M., Heeren T., Rose-Jacobs R., Cook J.T., Ettinger de Cuba S.A., Casey P.H., Chilton M. (2010). Development and validity of a 2-Item screen to identify families at risk for food insecurity. Pediatrics.

[86] Martin-Biggers J., Koenings M., Quick V., Abbot J.M., Byrd-Bredbenner C. (2015). Appraising nutrient availability of household food supplies using block dietary screeners for individuals. Eur. J. Clin. Nutr..

[87] Cheng C., Martin-Biggers J., Quick V., Byrd-Bredbenner C. (2016). Development of a parent-report questionnaire to evaluate physical activity availability, accessibility, and frequency in homes with preschool children. Int. J. Behav. Nutr. Phys. Act..

[88] Koszewski W., Behrends D., Nichols M., Sehi N., Jones G. (2011). Patterns of family meals and food and nutrition intake in limited resource families. Fam. Consum. Sci. Res. J..

[89] Neumark-Sztainer D., Story M., Hannan P.J., Croll J. (2002). Overweight status and eating patterns among adolescents: Where do youths stand in comparison to the Healthy People 2010 Objectives?. Am. J. Public Health.

[90] Neumark-Sztainer D., Story M., Hannan P.J., Perry C.L., Irving L.M. (2002). Weight-related concerns and behaviors among overweight and nonoverweight adolescents implications for preventing weight-related disorders. Arch. Pediatric Adolesc. Med..

[91] Neumark-Sztainer D., Wall M.M., Story M., Perry C.L. (2003). Correlates of unhealthy weight-control behaviors among adolescents: Implications for prevention programs. Health Psychol..

[92] Farmer M.M., Ferraro K.F. (2005). Are racial disparities in health conditional on socioeconomic status?. Soc. Sci. Med..

[93] Barbeau E.M., Krieger N., Soobader M.J. (2004). Working class matters: Socioeconomic disadvantage, race/ethnicity, gender, and smoking in NHIS 2000. Am. J. Public Health.

[94] Pampel F.C., Denney J.T., Krueger P.M. (2012). Obesity, SES, and Economic Development—A test of the Reversal Hypothesis. Soc. Sci. Med..

[95] Bartley M., Plewis I. (2002). Accumulated labour market disadvantage and limiting long-term illness: Data from 1971–1991 Office for National Statistics’ Longitudinal Study. Int. J. Epidemiol..

[96] Crissey S.R. (2009). Educational attainment in the United States: 2007.

[97] Drewnowski A., Darmon N. (2005). The economics of obesity: Dietary energy density and energy cost. Am. J. Clin. Nutr..

[98] Lovasi G.S., Hutson M.A., Guerra M., Neckerman K.M. (2009). Built environments and obesity in disadvantaged populations. Epidemiol. Rev..

[99] Molnar B.E., Gortmaker S.L., Bull F.C., Bulka S.L. (2004). Unsafe to play? Neighborhood disorder and lack of safety predict reduced physical activity among urban children and adolescents. Am. J. Health Promot..

[100] Campbell K.E., Marsden P.V., Hurlbert J.S. (1986). Social resources and socioeconomic status. Soc. Netw..

[101] Berkman L.F., Glass T. (2000). Social Integration, Social Networks, Social Support, and Health.

[102] Han S., Kim H., Lee H.S. (2012). A multilevel analysis of social capital and self-reported health: Evidence from Seoul, South Korea. Int. J. Equity Health.

[103] Mickelson K.D., Kubzansky L.D. (2003). Social distribution of social support: The mediating role of life events. Am. J. Community Psychol..

[104] Ljungvall A., Zimmerman F.J. (2012). Bigger bodies: Long-term trends and disparities in obesity and body-mass index among U.S. adults 1960–2008. Soc. Sci. Med..

[105] Abeyta I.M., Tuitt N.R., Byers T.E., Sauaia A. (2012). Effect of community affluence on the association between individual socioeconomic status and cardiovascular disease risk factors, Colorado, 2007–2008. Prev. Chronic Dis..

[106] Krieger N., Chen J.T., Waterman P.D., Soobader M.J., Subramanian S.V., Carson R. (2003). Choosing area based socioeconomic measures to monitor social inequalities in low birth weight and childhood lead poisoning: The Public Health Disparities Geocoding Project (US). J. Epidemiol. Community Health.

[107] Krieger N., Chen J.T., Waterman P.D., Soobader M.J., Subramanian S.V., Carson R. (2002). Geocoding and monitoring of US socioeconimic inequalities in mortality and cancer incidence: Does the choice of area-based measure and geographic level matter? The public health disparities geocoding project. Am. J. Epidemiol..

[108] Krieger N., Waterman P.D., Chen J.T., Soobader M.J., Subramanian S.V. (2003). Monitoring Socioeconomic Inequalities in Sexually Transmitted Infections, Tuberculosis, and Violence: Geocoding and Choice of Area-Based Socioeconomic Measures—The Public Health Disparities Geocoding Project (US). Public Health Rep..

[109] Charles C.Z. (2003). The dynamics of racial residential segregation. Annu. Rev. Sociol..

[110] Powell L.M., Chaloupka F.J., Bao Y. (2007). The availability of fast-food and full-service restaurants in the United States: Associations with neighborhood characteristics. Am. J. Prev. Med..

[111] Bleich S.N., Wang Y.C., Wang Y., Gortmaker S.L. (2009). Increasing consumption of sugar-sweetened beverages among US adults: 1988–1994 to 1999–2004. Am. J. Clin. Nutr..

[112] Agrawal T., Farrell T.J., Wethington E., Devine C.M. (2018). Doing our best to keep a routine: How low-income mothers manage child feeding with unpredictable work and family schedules. Appetite.

[113] Kelder S., Hoelscher D., Perry C., Glanz K., Rimer B., Viswanath K. (2015). How individuals, enviornments, and health behavior interact. Health Behavior, Theory, Research, and Practice.

[114] Woolf S.H., Aron L., National Research Council Committee on Population (2013). Physical and Social Environmental Factors. US Health in International Perspective: Shorter Lives, Poorer Health.

[115] Short S.E., Mollborn S. (2015). Social Determinants and Health Behaviors: Conceptual Frames and Empirical Advances. Curr. Opin. Psychol..

[116] Schulz M., Romppel M., Grande G. (2016). Built environment and health: A systematic review of studies in Germany. J. Public Health.

